# Prenatal Multivitamin Use and *MTHFR* Genotype Are Associated with Newborn Cord Blood DNA Methylation

**DOI:** 10.3390/ijerph17249190

**Published:** 2020-12-09

**Authors:** Kelly M. Bakulski, John F. Dou, Jason I. Feinberg, Katharine K. Brieger, Lisa A. Croen, Irva Hertz-Picciotto, Craig J. Newschaffer, Rebecca J. Schmidt, M. Daniele Fallin

**Affiliations:** 1 Department of Epidemiology, School of Public Health, University of Michigan, Ann Arbor, MI 48109, USA; bakulski@umich.edu (K.M.B.); johndou@umich.edu (J.F.D.); kbrieger@umich.edu (K.K.B.); 2 Department of Mental Health, Bloomberg School of Public Health, Johns Hopkins University, Baltimore, MD 21205, USA; jfeinbe2@jhu.edu; 3 Division of Research, Kaiser Permanente, Oakland, CA 94612, USA; Lisa.a.croen@kp.org; 4 Department of Public Health Sciences and the M.I.N.D. Institute, School of Medicine, University of California, Davis, CA 95616, USA; iher@ucdavis.edu (I.H.-P.); rjschmidt@ucdavis.edu (R.J.S.); 5 College of Health and Human Development, Penn State University, State College, PA 16802, USA; cjn5389@psu.edu

**Keywords:** DNA methylation, multivitamin, MTHFR, cord blood

## Abstract

Background: Fetal development involves cellular differentiation and epigenetic changes—complex processes that are sensitive to environmental factors. Maternal nutrient levels during pregnancy affect development, and methylene tetrahydrofolate reductase (*MTHFR*) is important for processing the nutrient folate. Hypothesis: We hypothesize that supplement intake before pregnancy and maternal genotype are associated with DNA methylation in newborns. Methods: In the pregnancy cohort, Early Autism Risk Longitudinal Investigation (EARLI), health history, and genotype information was obtained (n = 249 families). Cord blood DNA methylation (n = 130) was measured using the Illumina HumanMethylation450k array and global DNA methylation levels were computed over 455,698 sites. Supplement use preconception and during pregnancy were surveyed at visits during pregnancy. We evaluated associations between maternal preconception supplement intake and global DNA methylation or DNA methylation density distributions of newborn cord blood, stratified by the presence of a variant maternal *MTHFR* C677T allele. Results: Maternal preconceptional multivitamin intake was associated with cord blood methylation, dependent on maternal *MTHFR* genotype (interaction term *p* = 0.013). For mothers without the *MTHFR* variant allele, multivitamin intake was associated with 0.96% (95% CI: 0.09, 1.83) higher global cord blood methylation (*p* = 0.04) and was also associated with the cumulative density distribution of methylation (*p* = 0.03). For mothers with at least one variant allele, multivitamin intake had a null −0.06% (95% CI: −0.45, 0.33) association with global cord blood DNA methylation, and was not associated with the cumulative density distribution (*p* = 0.37). Conclusions: We observed that cord blood DNA methylation was associated with maternal supplement exposure preconception and maternal genotype. Genetic context should be considered when assessing DNA methylation effects of modifiable risk factors around the time of pregnancy.

## 1. Introduction

Pregnant mothers provide the nutrition source for a developing fetus. Many women take dietary supplements before or during pregnancy to augment dietary nutrition, in accordance with clinical recommendations [1]. The prevalence of use of one or more types of supplements during pregnancy is upwards of 90% in Europe and the United States [2] and 80% in China [3], however the periconception use of supplements is much lower, ranging from 0.9% to 50% globally [4].

Maternal intake of micronutrients is important in child development, impacting offspring health in a variety of ways. Maternal folate supplementation has clear impacts on fetal development. Folic acid is the synthetic form of folate contained in supplements as well as many multivitamins and prenatal vitamins. Low folate levels cause neural tube defects and periconceptional folic acid supplements can prevent up to 70% of neural tube defects [5]. Folate also impacts a range of early childhood developmental disorders. Maternal folic acid supplement intake before or during early pregnancy is associated with fewer childhood behavioral problems [6], improved verbal, verbal–executive function, attention, and social competence [7], fewer childhood hyperactivity and peer problems [8], and decreased risk for severe language delays [9]. Maternal use of prenatal vitamins in the first month of pregnancy is associated with reduced risk of autism spectrum disorder in the child [10].

Maternal *MTHFR* genotype impacts folate metabolism. Folate is metabolized through the one-carbon metabolism cycle and genetic factors impact cycling and availability of active byproducts to the fetus. The gene encoding the methylene tetrahydrofolate reductase (MTHFR) enzyme has a key role in converting folate to its biologically active form, 5-methyltetrahydrofolate. Common *MTHFR* genetic variants alter enzyme function and folate metabolism efficiency. At the *MTHFR* C677T locus, homozygous variant (TT) carriers have 70% lower enzyme activity and heterozygous variant (CT) carriers have 35% lower enzyme activity relative to those with the wild-type (CC) genotype [11]. As a consequence, those with the TT genotype have lower serum folate levels, and this difference can be eliminated with supplementation [12].

Folate is the primary methyl donor in the body, providing the substrate for nucleotide synthesis and DNA methylation [13]. Thus, DNA methylation levels may reflect overall one-carbon metabolism status. Indeed, maternal methyl-group intake is linked to infant DNA methylation. Long term folic acid supplementation before and during pregnancy is associated with higher candidate gene (*LEP* and *RXRA*) methylation in cord blood [14]. A meta-analysis of two cohorts found maternal plasma folate levels during pregnancy were associated with methylation levels of many CpG sites in newborn cord blood [15]. Maternal supplementation is associated with site specific DNA methylation levels, and our study seeks to investigate global DNA methylation in the context of pregnancy.

Maternal supplement intake and infant cord blood DNA methylation have never been investigated in relation to *MTHFR* genotype. In our study, we examine the associations between maternal supplement intake in the three months prior to pregnancy with infant cord blood DNA methylation. As folate is a key substrate for the one carbon metabolism cycle, which also feeds into methylation reactions, supplementation may have widespread impacts on the epigenome. Thus, we focus on whether there are global DNA methylation differences associated with preconceptional supplement use and we examine differences by *MTHFR* genotype.

## 2. Methods

### 2.1. Study Sample

In the Early Autism Risk Longitudinal Investigation (EARLI), mothers who had a previous child diagnosed with autism spectrum disorder were recruited into the pregnancy cohort [16]. Health history and biological samples were collected from the mothers, fathers, older probands with autism spectrum disorder, and baby siblings. Families were followed from pregnancy until the subsequent child was 36 months of age. Informed consent was obtained from all participants. The study was carried out in accordance with the Declaration of Helsinki, and was approved by Institutional Review Boards at all study sites: Johns Hopkins University (protocol number 00002032), Drexel University (protocol number 1201000673-R009), UC Davis (protocol number 225645-67), Kaiser Permanente (protocol number 00001045), and the secondary analysis site University of Michigan (protocol number HUM00115069).

### 2.2. Exposure Assessment

Supplement intake for each of 3 months prior to pregnancy was determined through surveys at enrollment. Mean gestational age at enrollment was 18.9 weeks (standard deviation 5.8). Mothers were asked whether they took multivitamins, folic acid, and prenatal vitamins in the 3 months before pregnancy. From their responses, the preconceptional supplement intake of mothers was categorized in a binary fashion by supplement type. We categorized mothers as taking preconceptional multivitamins if they reported use in any of the 3 months prior to pregnancy. Our primary exposure of interest was multivitamin intake. The same process was performed for the secondary exposures, which were folic acid supplements and prenatal vitamin intake.

### 2.3. Genetic Measures

Genetic data were measured using the Omni5+exome array (Illumina) at the John Hopkins University Center of Inherited Disease Research (CIDR). Data on 4,641,218 SNPs were generated for 841 EARLI family biosamples and 18 HapMap control samples. Note, the current paper analyzes associations with maternal and sibling genotypes, though genetic information was generated and preprocessed on all EARLI family members together, including fathers and probands. The sample concordance rate for duplicated HapMap samples was 99.72%, and for five blind duplicate samples the concordance rate was 99.9%. HapMap controls (n = 18), technical duplicates (n = 5), and samples from re-enrolled families (n = 9) were removed from analysis. No samples had missing genotypes at >3% of probes, or excess heterozygosity or homozygosity (4 standard deviations). Probes were removed if they had technical problems flagged by CIDR (n = 94,712) or missing genomic location information (n = 8124). Single nucleotide polymorphisms (SNPs) with minor allele frequencies >5% were removed if they had a missingness rate >5% (n = 8902), and SNPs with minor allele frequency <5% were removed if they had a missingness rate >1% (n = 65,855). Samples were merged with the 1000 genomes project (1000GP, version 5) data [17] and principal components were computed. EARLI measured genotype data were phased using SHAPEIT [18] and imputed to the 1000GP data using Minimac3 [19]. We kept SNPs with minor allele frequencies > 1%, leaving a total of 9,377,008 SNPs. We extracted measured genotype data for rs1801133, the site of the *MTHFR* C677T polymorphism. Mothers and children were categorized based on whether they had at least one measured “T” allele at rs1801133.

### 2.4. DNA Methylation Measures

Whole cord blood was collected at birth and stored frozen at −80 °C. DNA was extracted with the DNA Midi kit (Qiagen), then bisulfite treated and cleaned with the EZ DNA methylation gold kit (Zymo Research). Samples (n = 175) were assayed on the Infinium HumanMethylation450BeadChip (Illumina) at the John Hopkins University CIDR. DNA methylation arrays were run across two rounds.

### 2.5. DNA Methylation Data Processing

Raw image files were processed in R (version 3.5.1, Comprehensive R Archive Network, Auckland, New Zealand) statistical software with the minfi package [20]. Data were background and dye bias corrected with the normal-exponential using out-of-band probe (noob) method [21]. Cell type composition (granulocytes, monocytes, B-cells, CD8+ T-cells, CD4+ T-cells, and nucleated red blood cells) of cord blood samples were estimated from methylation using a sorted cord blood cell reference [22]. Probes were dropped from analysis if they were mapped to sex chromosomes (n = 11,648), failed detection *p*-value of >0.01 in >10% of samples (n = 661) or were documented as cross reactive (n = 29,233) [23]. A final count of 445,241 probes remained in analysis. Mean DNA methylation per person was calculated as the mean across all probes. Mean DNA methylation restricted to probes in genomic regions (CpG island, shore, shelf, or open sea) were also computed. Genomic region assignment was based on Illumina’s annotation of CpG sites.

### 2.6. Study Sample Descriptive Statistics

Analysis excluded cord blood samples with discordant DNA methylation estimated sex and observed sex (n = 3) and non-singleton births (n = 2). We further limited the analysis set to mother/child pairs with complete data on supplement use, genetics, and covariates of interest, resulting in an additional 40 dropped samples (23%). Our study sample with complete data included 130 mother–child pairs. Covariates considered were batch, sex, maternal age, and maternal education. We described our sample using univariate statistics (mean and standard deviation for continuous covariates; number and frequency for categorical variables). In tables, we provide counts for self-report maternal race, but in analysis we used principal components for genetic ancestry adjustment. We computed bivariate relationships between supplement usage preconception with mean DNA methylation and covariates (t-tests for mean DNA methylation and other continuous covariates, chi-square test for categorical covariates).

### 2.7. Single Site DNA Methylation Analysis

We examined differential methylation at single sites by fitting linear models at each probe. Single site models used supplement intake preconception as a binary exposure and noob corrected beta DNA methylation values as the outcome. Models were adjusted for infant sex, laboratory processing round, genetic ancestry principal components, and cell type (nucleated red blood cell and granulocyte estimated proportions). We used empirical Bayes standard error moderation in the limma package [24]. We fit the models for each supplement independently. The reference group for these models were mothers who did not take any of the three supplements preconception (n = 41 mothers). We computed lambda inflation values for each model. We also fit models for each supplement stratified by maternal *MTHFR* C677T status. We visualized single site results by creating volcano plots of estimated effect sizes and −log *p*-values. We accounted for multiple comparisons using false discovery rate (FDR) calculations.

We conducted similar analysis for the effect of *MTHFR* C677T on DNA methylation. For these models, the CC genotype was the reference group. We did one model investigating the effect of maternal *MTHFR* status on cord blood DNA methylation, adjusted for the same covariates as in the supplement models, and another assessing the effect of child *MTHFR* status on cord blood DNA methylation. Additionally, we computed Pearson correlations of single site effect estimates of the two *MTHFR* models. We also computed correlations between the supplement models, and *MTHFR* compared to supplements.

We tested for enrichment in the CpGs with *p*-value < 0.001 for gene ontology biological processes using the missMethyl package [25]. This was completed for each of the single site supplement results and *MTFHR* results. Gene ontology terms were restricted to those containing a minimum of five observed genes. REVIGO (Rudjer Boskovic Institute, Zagreb, Croatia) was used to condense redundant gene ontologies [26].

### 2.8. Global Methylation Analysis

We compared differences in mean DNA methylation by preconceptional supplement usage, and by presence of *MTHFR* C677T polymorphism. We first fit linear models with mean DNA methylation as the outcome and supplement use as the exposure. We fit separate models for each of the supplements: multivitamins, folic acid, and prenatal vitamins. The reference group for each supplement model was the 41 mother/child pairs with no preconceptional supplement use. Models were adjusted for sex, laboratory processing round, ancestry principal components, maternal age, maternal education, and cell type proportions (nucleated red blood cell and granulocyte estimated proportions). We next stratified the supplement use models based on presence of the *MTHFR* C677T polymorphism to see whether the beta term for a supplement’s effect was different. To test for effect modification by maternal *MTHFR* we fit an adjusted model with an interaction term for supplement and maternal *MTHFR* status. In total, one interaction model was fit for each of the three supplements.

As a sensitivity analysis, we examined global DNA methylation with the Global Analysis of Methylation Profiles (GAMP) package. This method tests for differences in the cumulative distribution functions of probe methylation values [27]. For both the mean DNA methylation and GAMP methods, our primary models conducted tests using all probes. We also conducted tests restricting to CpG islands, shores, shelves, and open sea regions.

### 2.9. Tissue Specificity

In addition to cord blood, we also examined preconceptional supplement and global DNA methylation patterns in other tissues. Collection and DNA methylation measures were also performed for maternal blood in early pregnancy (n = 201) and late pregnancy (n = 118), and placenta on the fetal side (n = 134) and maternal side (n = 132). We fit models, stratified by maternal *MTHFR*, in maternal blood and placenta data. Maternal blood models were adjusted for maternal education, maternal age, ancestry principal components, and granulocyte cell proportion. Placenta models were adjusted for maternal education, maternal age, ancestry principal components, and sex of child. After dropping for missingness, early pregnancy maternal blood had n = 154 and late pregnancy maternal blood had n = 84. In fetal side placenta we had nonmissing n = 102, and in maternal side placenta n = 105. Analyses were performed as described for cord blood.

## 3. Results

### 3.1. Study Sample Characteristics

A total of 130 mother/child pairs had data on cord blood DNA methylation and all variables of interest (preconceptional supplement intake, sex, *MTHFR* genotype, maternal age, and maternal education). In the 3 months prior to pregnancy, 44 (33.8%) mothers reported taking multivitamin supplements. A smaller number of mothers (n = 15, 11.5%) reported folic acid use. Prenatal vitamins were used prior to pregnancy by 51 (39.2%) mothers. At least one copy of the *MTHFR* C677T polymorphism was present in 48.5% of the offspring, and in 57.7% of mothers (Supplementary Table S1). Our study sample was highly educated (66.1% with 4 years of college or more), older (mean maternal age of 33.62 years), and about half (50.8%) were non-Hispanic white.

Multivitamin supplement intake prepregnancy did not differ by covariates. However, mothers with a copy of the *MTHFR* C677T variant were more likely to take multivitamins (65.9% versus 53.5%, *p* = 0.24) (Table 1). Bivariate results by preconceptional folic acid (Supplementary Table S2) and prenatal vitamin (Supplementary Table S3) intake were similar. Prenatal vitamin usage preconception was associated with maternal education. Most (78.4%) mothers who took prenatal vitamins preconception had at least 4 years of college, while 58.3% of mothers who did not take prenatal vitamins had that level of education. Child *MTHFR* status and maternal *MTHFR* status were correlated (*p* < 0.001). *MTHFR* status was also associated with maternal race (*p* = 0.006). Mothers with a variant *MTHFR* allele were 58.7% non-Hispanic white, while mothers that were wild type CC genotype were 40% non-Hispanic white (Table 2).

### 3.2. Supplement Intake on Single Site DNA Methylation

**Figure 1:**
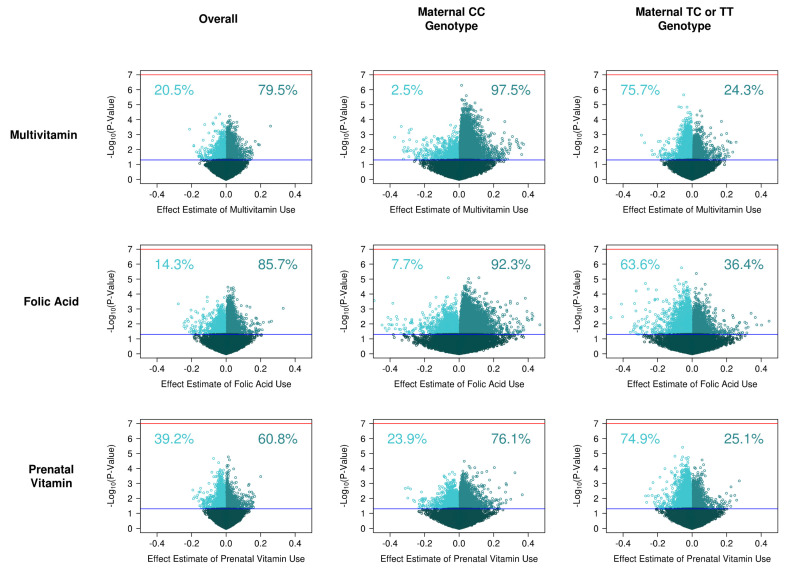
Association between maternal supplement use in the 3 months prior to conception and cord blood DNA methylation. Each dot represents a CpG site measured on the array. Percentages of nominal (*p* < 0.05) CpGs that are hypo/hyper methylated are shown.

No individual CpG site reached genome-wide significance for association with supplement use pre-pregnancy (Figure 1). The model for single site DNA methylation in relation to multivitamin use had a lambda inflation factor of 1.26 (Supplementary Figure S1A). In relation to multivitamin use, three CpGs had a *p*-value < 10^−4^. Multivitamin use was associated with a −3.7% decrease in methylation at cg03665785 (annotated to *C22orf31*), 2.0% increase in methylation at cg11332951, and a −6.0% decrease at cg05877788 (annotated to *TP53I13*).

The model testing associations with preconception folic acid supplement use had lambda of 1.37 (Supplementary Figure S1B). Importantly, few mothers (n = 15) reported preconception folic acid intake and these findings should be considered exploratory. A total of 7 CpGs had *p*-value < 10^−4^ in relation to folic acid supplement use. Preconception folic acid use was associated with a 1.1% increase in methylation at cg05701403 (*PLEKHA7*), 4.2% increased methylation at cg25490527 (*ANO1*), 2.9% increase at cg04927695 (*KCNT1*), 3.2% increase at cg17311726, 4.7% increase at cg23065768 (*KCNJ11*), 1.4% increase at cg04121214, and a 4.0% increase at cg06102690 (*CCDC149*).

The prenatal vitamins model had lambda of 0.80 (Supplementary Figure S1C). Seven CpGs had *p*-value < 10^−4^ in relation to preconceptional prenatal vitamin use. Prepregnancy prenatal vitamin use was associated with 1.3% increased methylation at cg12869912, −7.2% decreased methylation at cg01587386, 1.9% increase at cg19585156 (*IGSF21*), −4.3% decrease at cg24757159 (*MIR548G*), 1.2% increase at cg12666279 (*DPP10*), 0.51% increase at cg07104706 (*SLITRK1*), and 0.6% increase at cg05118419 (*TBCEL*).

Effect estimates for the supplement models were correlated. Single site estimates for the multivitamin model and folic acid model had Pearson correlation r = 0.58. Multivitamin effect estimates and prenatal vitamin effect estimates had correlation r = 0.51. Lastly, the prenatal vitamin and folic acid single site effect estimates had correlation r = 0.42.

### 3.3. MTHFR and Single Site DNA Methylation

No individual CpG site had genome-wide significance with *MTHFR* C677T status. The model for child *MTHFR* C677T had considerable inflation with lambda of 2.54 (Supplementary Figure S1D). In relation to child *MTHFR* C677T status, 12 CpGs reached *p*-value < 10^−5^. The top site was cg05962522, where having the variant allele was associated with 1.7% higher methylation (*p*-value = 1.2 × 10^−6^). The single site maternal *MTHFR* model also had considerable inflation with lambda of 2.44 (Supplementary Figure S1E). In relation to maternal *MTHFR* C677T status, 14 CpGs reached *p*-value < 10^−5^. The top CpG was cg20399131 (mapped to *C2orf78*) where presence of the variant allele was associated with 2.4% higher methylation (*p*-value = 1.09 × 10^−6^).

The Pearson correlation of single DNA methylation site effect estimates between the maternal *MTHFR* and the child *MTHFR* models was r = 0.29. DNA methylation site effect estimates of preconception multivitamin intake were correlated with DNA methylation site effect estimates for maternal *MTHFR* (r = 0.36) and child *MTHFR* (r = 0.37) genotypes.

### 3.4. Supplement Intake on Single Site DNA Methylation, Stratified by Genotype

Of the CpG sites with nominal association (*p*-value < 0.05), 79.5% showed higher methylation associated with multivitamin use. We also fit models stratified by maternal *MTHFR* C677T genotype. For wild type mothers (CC genotype) 97.5% of CpGs nominally associated with multivitamins were hypermethylated. For mothers with a variant allele (CT or TT genotypes) 24.3% of nominal CpGs were hypermethylated (Figure 1). Stratified by child *MTHFR* C677T genotype there was a similar, but less extreme, pattern. In children with CC genotype, 90.2% of nominal CpGs were hypermethylated. In children with CT or TT genotype, 42.4% were hypermethylated (Supplementary Table S4).

For folic acid use, 85.7% of nominally associated CpGs were hypermethylated. As in the case of multivitamins, higher percentages of nominal CpG sites were hypermethylated when mothers had the wild type *MTHFR* 677 CC genotype (92.3%) than in mothers with a variant CT or TT genotype (36.4%). In children with CC genotype, 81.1% of nominal CpGs were hypermethylated. In children with CT or TT genotype, 42.4% was hypermethylated.

For prenatal vitamin use 60.8% of nominal CpGs were hypermethylated. When the mother had CC genotype 76.1% of nominal CpGs were hypermethylated. When mothers had CT or TT genotype, 25.1% of nominal CpGs were hypermethylated. Stratified by child *MTHFR* C677T genotype, the opposite pattern was observed. There was more hypermethylation in children with CT or TT genotype, with 65.2% of nominal CpGs. In children with CC genotype, 49.8% of nominal CpGs had hypermethylation.

### 3.5. Pathway Analysis

Gene ontology enrichment in the top DNA methylation sites (CpGs with *p* < 0.001) was completed for each supplement. The top biological process term for multivitamin use was homophilic cell adhesion via plasma membrane adhesion molecules (Supplementary Table S5) (*p* = 1.9 × 10^−11^, FDR = 4.24 × 10^−7^). No other pathway had FDR < 1.0. The top ontology for folic acid was regulation of small GTPase mediated signal transduction (*p* = 0.0006, FDR = 1.0). None of the top pathways with prenatal vitamins had greater than five observed gene terms, so instead we report terms with greater than two observed terms. The highest ranked biological process with prenatal vitamins was morphogenesis of embryonic epithelium (*p* = 0.003, FDR = 1.0).

Child *MTHFR* single site results were enriched for the positive regulation of cell-substrate adhesion pathway (*p* = 8.8 × 10^−7^, FDR = 0.01) (Supplementary Table S6). In maternal *MTHFR* model enrichment results, the cell–substrate adhesion pathway was also among the top pathways (*p* = 2.3 × 10^−4^, FDR = 0.58). The top pathway for maternal *MTHFR* was cell junction assembly (*p* = 4.6 × 10^−5^, FDR = 0.23). In common with the top folic acid pathway, regulation of small GTPase mediated signal transduction was also a top enriched pathway for maternal *MTHFR* related CpG sites (*p* = 6.15 × 10^−5^, FDR = 0.23).

### 3.6. Supplement Intake Associations with Global DNA Methylation

Mean cord blood DNA methylation was higher when mothers took multivitamin supplements preconception. The overall unadjusted effect for multivitamin intake was 0.31% (95% CI: −0.06, 0.69) higher cord blood methylation (*p* = 0.11). When adjusting for sex, maternal age, maternal education, ancestry principal components, batch, and cell type proportions, the effect of multivitamins on mean overall DNA methylation was a 0.20% (95% CI: −0.17, 0.58) increase (*p* = 0.29). The largest difference was in the shelf region, with multivitamin use associated with 0.34% (95% CI: −0.23, 0.91) higher methylation (*p* = 0.24) (Supplementary Figure S2). The lowest difference was seen in the island region where multivitamin use was associated with 0.08% (95% CI: −0.16, 0.32) higher methylation (*p* = 0.53).

Folic acid intake had effects similar in magnitude to multivitamins, though wider confidence intervals. Unadjusted, folic acid use preconception had 0.31% (95% CI: −0.21, 0.83) higher methylation (*p* = 0.25) than the no supplement group. The adjusted effect of folic acid use was a 0.38% (95% CI: −0.12, 0.88) increase (*p* = 0.14) in overall DNA methylation. The largest adjusted difference was in the shore region, where the folic acid supplement group had 0.60% (95% CI: −0.20, 1.39) higher methylation (*p* = 0.15). Similar to multivitamins, the island region had the smallest change, where folic acid intake was associated with 0.20% (95% CI: −0.09, 0.49) increased methylation (*p* = 0.19).

Prenatal vitamin intake preconception had much weaker associations than multivitamins or folic acid. Unadjusted, the prenatal vitamin group had 0.02% (95% CI: −0.38, 0.43) higher overall global methylation (*p* = 0.91). Following adjustment for covariates, the effect of prenatal vitamins was a −0.09% (95% CI: −0.50, 0.33) decrease (*p* = 0.68).

### 3.7. Supplement Intake Associations with Global DNA Methylation, Stratified by Genotype

**Figure 2:**
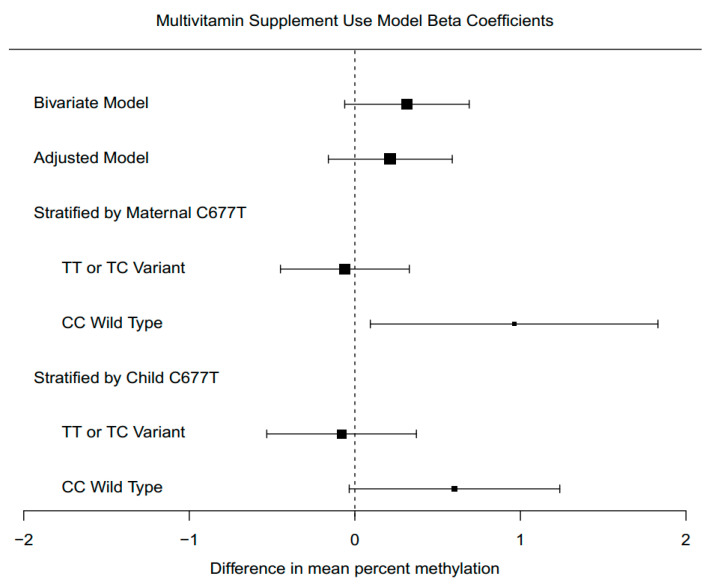
Estimated model coefficients for effect of prepregnancy multivitamin use. Models were adjusted for sex, maternal age, maternal education, ancestry, batch, and cell type proportions for nucleated red blood cells and granulocytes.

The associations between preconception supplement intake and global DNA methylation were modified by the presence of a *MTHFR* variant allele. We observed an interaction between *MTHFR* genotype and multivitamin use on global DNA methylation (interaction *p* = 0.013). In stratified analyses, for mothers with the wild-type genotype, multivitamin supplementation was associated with 0.96% (95% CI: 0.09, 1.83) higher cord blood methylation (*p* = 0.042), after adjustment. In mothers with a TT or TC genotype, the effect estimate was a −0.06% (95% CI: −0.45, 0.33) change (*p* = 0.76) (Figure 2).

We observed a marginal interaction between *MTHFR* 677 genotype and folic acid intake on global DNA methylation (*p* = 0.062). For folic acid supplement use, the difference in global methylation was also greater when mothers had the CC genotype (Supplementary Figure S3). For mothers with the CC genotype, we observed a 1.24% (95% CI: −0.08, 2.58) increase in global cord blood methylation. When mothers had the variant TT or TC genotype, folic acid use was associated with a 0.15% (95% CI: −0.37, 0.66) increase in cord blood methylation.

We did not observe an interaction between *MTHFR* 677 genotype and prenatal vitamins on global DNA methylation (*p* = 0.44). When stratified by *MTHFR* 677, for CC genotype mothers, prenatal vitamin supplementation was associated with a 0.11% (95% CI: −0.88, 1.11) increase in cord blood methylation. When mothers had the variant allele, prenatal vitamins were associated with a −0.20 (95% CI: −0.68, 0.29) decrease in cord blood methylation (Supplementary Figure S4).

Findings using GAMP to test differences in the shape of methylation distributions by multivitamin use mirrored results using mean DNA methylation. Cumulative density distribution (CDF) differences were significantly associated with multivitamin use unadjusted (*p* = 0.023), but not when adjusted (*p* = 0.11). When stratifying by maternal *MTHFR* C677T and adjusting for covariates, the CDF was associated with multivitamin use in the maternal wild type group (*p* = 0.029), but not associated in the TT or TC variant group (*p* = 0.37) (Supplementary Table S7).

### 3.8. Tissue Specificity

We also explored associations of global methylation and preconceptional multivitamin intake in maternal blood and placenta, in relation to *MTHFR* C677T status. There were no significant differences by multivitamin use, regardless of genotype in these tissues (Supplementary Table S8).

## 4. Discussion

In this study, preconceptional multivitamin intake was associated with global DNA methylation patterns. We found that this relationship had a significant interaction with maternal *MTHRF* C677T status. If mothers did not have the variant allele, multivitamin use was associated with a 0.96% (95% CI: 0.09, 1.83) increase in offspring cord blood methylation (*p* = 0.042). If mothers did have a copy of the T allele, then there was no difference in infant cord blood methylation (*p* = 0.76). We observed a similar pattern with folic acid supplementation, with larger effects when mothers do not have the *MTHFR* variant allele, though with relatively few participants taking folic acid in this study sample, these folic acid findings should be interpreted cautiously.

The relationship between maternal nutritional exposures and infant DNA methylation has been inconsistent, with considerable variation depending on timing, type of exposure measures, and other factors [28]. In general, across micronutrient studies on arrays, there has been minimal replication of findings [29]. For example, previous study of maternal plasma folate observed associations with infant cord blood DNA methylation [15], however only 2 CpGs achieved *p*-value < 0.001 in that study and our study: cg02967610 and cg14116463. Though specific site overlap with this previous study was minimal, our pathway results showed similar types of enriched pathways, namely nervous system development and cell signaling. In addition, we found cell adhesion pathways related to supplements and *MTHFR*. Cell–cell adhesion plays an important role in neural plate formation [30]. Enrichment of the small GTPase mediate signal transduction pathway may also be important, since small GTPases regulate cell protrusions necessary for neural tube closure [31]. Though they need to be interpreted with caution due to liberal *p*-value cutoffs employed, the enriched pathways reinforce the relevance of supplementation to neural tube defects and early neurodevelopment.

Based on these findings, it is likely that higher availability of methyl donors has stronger global effects on DNA methylation, as opposed to site specific regulation. With respect to global DNA methylation, periconceptional choline intake in the second trimester was inversely associated with LINE-1 DNA methylation in cord blood of male offspring, with no other associations found with maternal intake of other methyl donors, such as folate and vitamin B12 [32]. On the other hand, higher levels of maternal choline intake in the third trimester was associated with increased global placental methylation, but not significantly maternal blood or cord blood methylation [13]. In maternal blood and placenta, we did not observe global differences in DNA methylation related to supplementation. In placenta, the direction and patterns with *MTHFR* C677T held however, with positive trends with preconceptional supplementation and a lower or opposite trend with the C677T variant. Our findings show some overlap with prior reports, however there is great heterogeneity in exposure and DNA methylation methodological approaches implemented, including adjustment for cell heterogeneity.

Measures of exposure may include direct serum or plasma levels, or report of supplement intake (as in the current study). Measurements of exposure could also be taken at different time points during pregnancy or prior to pregnancy, which may result in different patterns of methylation and play a role in variability across studies. The earliest period may be especially influential, since following fertilization rapid de-methylation occurs, and is re-established through development [33]. Global DNA methylation and hydroxymethylation levels in maternal blood vary throughout pregnancy and in the third trimester, higher methyl-group intake is associated with concurrent higher maternal blood DNA methylation [34]. There may also be differences in tissue types or timing measured for DNA methylation. Previously in an animal study, folic acid supplementation was associated with decreased brain global DNA methylation (measured by in vitro DNA methyl acceptance capacity) with gestation period dependent effects, but did not change kidney and colon DNA methylation [35].

Genetic variability is important when considering the relationship between folic acid supplementation on DNA methylation. Previous research observed that the *MTHFR* C677T TT genotype and low folate status was associated with blood hypomethylation, relative to the CC genotype [36]. Global DNA methylation was also found to be lower in women with the TT genotype, relative to the CT or CC genotype, after a period of folate restriction followed by folate treatment [37]. In a randomized trial of folic acid supplementation in nonpregnant women, folic acid supplementation followed by withdrawal is associated with decreased global methylation, with the largest decrease in the CC genotype group [38]. In contrast, in adult males, high dose folic acid supplementation was associated with lower sperm DNA methylation, and global methylation differences were largest in the TT genotype group [39]. More recently, analysis of publicly available data (GSE74548) on the association between long-term daily folic acid and vitamin B12 supplementation in elderly subjects with global DNA methylation, also using the 450k array and defining global methylation in the same way, observed a significant interaction between supplementation and *MTHFR* genotype, where increased methylation was observed in the CC genotype group and not in the TT genotype group [40]. Our findings for maternal preconceptional supplementation and cord blood DNA methylation indicate a pattern dependent upon *MTFHR* C677T consistent with past research on supplementation and methylation in blood.

Our study was limited by several contributing factors. One caveat is that we defined global DNA methylation as the mean over hundreds of thousands of measures on the array. This proxy measure, and others such as DNA methylation at repeat elements, may not necessarily accurately reflect genome-wide global DNA methylation levels, and care should be taken when considering different measures and contexts [41]. Effect estimates for the associations between folic acid and DNA methylation were similar in magnitude to the multivitamins. However, our sample size of mothers taking folic acid preconception was small (n = 15), so as expected we observed with precision in the folic acid effect estimates. It is possible an association with folic acid supplement intake would be detected if the study had a larger number of folic acid users. Another consideration is the timing of supplement use during pregnancy. Prenatal vitamin use increased dramatically during pregnancy in our study, with >80% taking prenatal vitamins by month 4 of pregnancy. We observed largely null associations between preconception prenatal vitamin intake and cord blood DNA methylation, though the biologic basis of this observation requires further study. If prenatal vitamin use during pregnancy had impacts on cord blood DNA methylation, it might have washed out potential effects of prenatal vitamin intake preconception.

The biological importance of the observed magnitude of difference (0.96% higher DNA methylation in cord blood in infants of mothers without the *MTHFR* variant who took multivitamins) is unclear. Biologically, we would expect widespread tissue differences in DNA methylation related to normal cellular differentiation [42,43], with placenta having among the most distinct DNA methylation patterns of all tissues [44]. Put in the context of tissue differences within our own data, we observed cord blood DNA methylation had 6.3% higher global DNA methylation compared to placenta tissue. Together, this suggests that in our study the magnitude of the genetic and environmental exposure association with global DNA methylation was 15% of the magnitude of tissue differences in global DNA methylation. We also compared the magnitude of difference observed in our study to the magnitude of DNA methylation differences reported with other environmental exposures, though the DNA methylation measures and comparisons implemented vary. For example, a doubling in prenatal red blood cell mercury concentration was associated with 0.031% lower global 5-hydroxymethylcytosine in early childhood blood [45]. Exposure to polycyclic aromatic hydrocarbon compounds/metabolites in the third tertile relative to the first tertile of the study sample was associated with 1-2% lower Alu/Line-1 global DNA methylation levels [46]. For every unit (μg/m3) increase in nitrogen oxide exposure, somatic chromosome global DNA methylation was 0.01% lower in EPIC-Italy [47]. Relative to tissue differences and prior environmental exposure differences in global DNA methylation, the magnitude of difference in DNA methylation observed is our study is meaningful, however the downstream consequences of this global genetic and environmental exposure related DNA methylation differences remain to be seen.

Adequate maternal nutritional status is essential for proper fetal development. Maternal supplementation has been instrumental in reducing the population burden of neural tube defects and other developmental abnormalities. Maternal multivitamin supplementation and folic acid use before or during pregnancy are similarly associated with reduced autism risk in offspring [48]. Understanding the epigenetic effects of this supplementation, and the relationships across genetic variant groups, are essential. In the current study, maternal supplement use was assessed prospectively and multiple types of supplements were queried. Maternal and child genotypes were also measured and infant cord blood DNA methylation was quantitated. Joint consideration of genetics and environmental exposures is relatively rare in DNA methylation studies and this study demonstrates the importance. We observed that maternal supplement use was associated with increased cord blood DNA methylation, only among wildtype *MTHFR* genotype carriers. Measuring environmental and genetic specific molecular consequences during pregnancy is essential for understanding development.

## Figures and Tables

**Table 1:** Multivitamin use preconception by covariates in the Early Autism Risk Longitudinal Investigation (EARLI). *p*-values for difference in variables by multivitamin intake is from t-test for continuous variables, and chi-square test for categorical variables. Acronyms: Standard deviation (sd), methylenetetrahydrafolate reductase gene (*MTHFR*), cytosine (C), thymine (T).

	Study Sample (n = 130)	Multivitamin Use Preconception		*p*-Value
		No (n = 86)	Yes (n = 44)	
**Child/Cord Blood Characteristics**				
DNA Methylation (mean, sd)				
Overall	50.80 (0.94)	50.7 (1.0)	51.0 (0.9)	0.05
Open Sea	73.61 (1.30)	73.4 (1.4)	74.0 (1.1)	0.02
Shelf	78.06 (1.40)	77.9 (1.5)	78.4 (1.2)	0.018
Shore	46.76 (0.87)	46.7 (0.8)	47.0 (0.9)	0.061
Island	19.40 (0.58)	19.4 (0.6)	19.4 (0.6)	0.59
Cell Type Percent (mean, sd)				
Nucleated red blood cells	55 (42.3%)	10.1 (5.3)	9.14 (5.0)	0.30
Granulocytes	75 (57.7%)	43.5 (11.6)	43.3 (13.8)	0.96
Methylation Measurement Round				0.58
1	63 (48.5%)	59 (68.6%)	33 (75.0%)	
2	67 (51.5%)	27 (31.4%)	11 (25.0%)	
Sex				0.16
Female	64 (49.2%)	38 (44.2%)	26 (59.1%)	
Male	66 (50.8%)	48 (55.8%)	18 (40.9%)	
Child *MTHFR* C677T Genotype				0.76
CC	63 (48.5%)	43 (50.0%)	20 (45.5%)	
TT or TC	67 (51.5%)	43 (50.0%)	24 (54.5%)	
**Maternal Characteristics**				
Age (years)	33.62 (4.63)	33.4 (4.4)	34.0 (5.1)	0.48
Education				0.66
High School or Less	26 (20.0%)	19 (22.1%)	7 (15.9%)	
Less Than 4 Years College	18 (13.8%)	10 (11.6%)	8 (18.2%)	
4 Years of College	57 (43.8%)	37 (43.0%)	20 (45.5%)	
Post Graduate	29 (22.3%)	20 (23.3%)	9 (20.5%)	
Race/Ethnicity				0.99
Non-Hispanic White	66 (50.8%)	43 (50.0%)	23 (52.3%)	
Non-Hispanic Black	12 (9.2%)	8 (9.3%)	4 (9.09%)	
Hispanic/Latino	14 (10.8%)	10 (11.6%)	4 (9.09%)	
Other	38 (29.2%)	25 (29.1%)	13 (29.5%)	
Folic Acid Use Preconception				0.81
No	115 (88.5%)	77 (89.5%)	38 (86.4%)	
Yes	15 (11.5%)	9 (10.5%)	6 (13.6%)	
Prenatal Vitamin Use Preconception				0.001
No	79 (60.8%)	43 (50.0%)	36 (81.8%)	
Yes	51 (39.2%)	43 (50.0%)	8 (18.2%)	
Maternal *MTHFR* C677T Genotype				0.24
CC	55 (42.3%)	40 (46.5%)	15 (34.1%)	
TT or TC	75 (57.7%)	46 (53.5%)	29 (65.9%)	

**Table 2:** *MTHFR* variant status by covariates in the Early Autism Risk Longitudinal Investigation (EARLI). *p*-values for difference in variables by multivitamin intake is from t-test for continuous variables, and chi-square test for categorical variables. Acronyms: Standard deviation (sd), methylenetetrahydrafolate reductase gene (*MTHFR*), cytosine (C), thymine (T), nucleated red blood cell (nRBC).

	*MTHFR* C677T Allele Present in Child			*MTHFR* C677T Allele Present in Mother		
	No (n = 63)	Yes (n = 67)	*p*-Value	No (n = 55)	Yes (n = 75)	*p*-Value
**Child/Cord Blood Characteristics**						
DNA Methylation (mean, sd)						
All	50.6 (1.04)	50.9 (0.81)	0.07	50.7 (1.14)	50.9 (0.75)	0.24
Open Sea	73.4 (1.52)	73.8 (1.02)	0.045	73.4 (1.67)	73.8 (0.93)	0.16
Shelf	77.8 (1.62)	78.3 (1.12)	0.038	77.9 (1.78)	78.2 (1.03)	0.17
Shore	46.6 (0.92)	46.9 (0.80)	0.124	46.7 (1.00)	46.8 (0.75)	0.46
Island	19.3 (0.54)	19.4 (0.61)	0.342	19.4 (0.58)	19.4 (0.58)	0.58
Cell Type Percent (mean, sd)						
nRBC	9.84 (5.0)	9.74 (5.4)	0.91	10.1 (5.9)	9.54 (4.6)	0.54
Granulocytes	43.7 (12.8)	43.2 (11.9)	0.82	44.1 (12.5)	42.9 (12.2)	0.58
DNA Methylation Round			0.97			0.58
1	44 (69.8%)	48 (71.6%)		37 (67.3%)	55 (73.3%)	
2	19 (30.2%)	19 (28.4%)		18 (32.7%)	20 (26.7%)	
Child Sex			0.87			0.39
Female	32 (50.8%)	32 (47.8%)		30 (54.5%)	34 (45.3%)	
Male	31 (49.2%)	35 (52.2%)		25 (45.5%)	41 (54.7%)	
Child *MTHFR* C677T	-	-	-			<0.001
CC Genotype				41 (74.5%)	22 (29.3%)	
TT or TC Genotype				14 (25.5%)	53 (70.7%)	
**Maternal Characteristics**						
Age (years)	33.9 (4.50)	33.4 (4.76)	0.58	33.9 (4.3)	33.4 (4.9)	0.57
Multivitamins			0.63			0.19
None Pre- Conception	43 (68.3%)	42 (62.7%)		40 (72.7%)	45 (60.0%)	
Used Pre- Conception	20 (31.7%)	25 (37.3%)		15 (27.3%)	30 (40.0%)	
Folic Acid			0.56			0.61
None Preconception	55 (87.3%)	55 (82.1%)		45 (81.8%)	65 (86.7%)	
Used Preconception	8 (12.7%)	12 (17.9%)		10 (18.2%)	10 (13.3%)	
Prenatal Vitamins			0.41			0.37
None Preconception	35 (55.6%)	43 (64.2%)		30 (54.5%)	48 (64.0%)	
Used Pre-conception	28 (44.4%)	24 (35.8%)		25 (45.5%)	27 (36.0%)	
Education			0.63			0.53
High school or less	12 (19.0%)	14 (20.9%)		9 (16.4%)	17 (22.7%)	
<4 Yrs College	9 (14.3%)	9 (13.4%)		10 (18.2%)	8 (10.7%)	
4 Yrs of College	25 (39.7%)	32 (47.8%)		25 (45.5%)	32 (42.7%)	
Post Graduate	17 (27.0%)	12 (17.9%)		11 (20.0%)	18 (24.0%)	
Race/Ethnicity			0.002			0.006
Non-Hispanic White	26 (41.3%)	40 (59.7%)		22 (40.0%)	44 (58.7%)	
Non-Hispanic Black	11 (17.5%)	1 (1.49%)		10 (18.2%)	2 (2.67%)	
Hispanic/Latino	4 (6.35%)	10 (14.9%)		4 (7.27%)	10 (13.3%)	
Other	22 (34.9%)	16 (23.9%)		19 (34.5%)	19 (25.3%)	
Maternal *MTHFR* C677T			<0.001	-	-	-
CC Genotype	41 (65.1%)	14 (20.9%)				
TT or TC Genotype	22 (34.9%)	53 (79.1%)				

## Supplementary Materials

The following are available online at https://www.mdpi.com/1660-4601/17/24/9190/s1, Table S1: Full sample with cord blood DNA methylation measures and study sample after removing for missingness; Table S2: Folic acid use preconception by covariates; Table S3: Prenatal vitamin use preconception by covariates; Table S4: Among DNA methylation sites nominally associated (*p* < 0.05) with each exposure and genotype, number and proportion of sites that were hypermethylated and hypomethylated; Table S5: Top pathways for single site supplement models; Table S6: Top pathways for single site MTHFR C677T models; Table S7: GAMP test *P*-values for the effect of preconception supplement intake on cumulative density distribution of cord blood DNA methylation; Table S8: Global methylation differences by multivitamin supplementation prior to pregnancy, stratified by MTHFR genotype, in maternal blood and placenta; Figure S1: QQ-plots of single site models; Figure S2: Unadjusted supplement and global DNA methylation by genomic regions; Figure S3: Estimated model coefficients for effect of preconception folic acid use; Figure S4: Estimated model coefficients for effect of preconception prenatal vitamin use.

## Acronyms

Confidence interval (CI), John Hopkins University Center of Inherited Disease Research (CIDR), cytosine nucleotide phosphate bonded to a guanine nucleotide (CpG), Early Autism Risk Longitudinal Investigation (EARLI), false discovery rate (FDR), methylene tetrahydrofolate reductase (*MTHFR*), single nucleotide polymorphism (SNP).
