# Supplementary material for: Prenatal Multivitamin Use and MTHFR Genotype Are Associated with Newborn Cord Blood DNA Methylation
Source: Int J Environ Res Public Health. 2020 Dec 9;17(24):9190. doi: 10.3390/ijerph17249190 (PMC7764679; doi:10.3390/ijerph17249190)

Supplementary Information:

This document contains 8 supplementary tables and 4 supplementary figures.

Supplementary Table S1: Full sample with cord blood DNA methylation measures and study sample after removing for missingness. P-values were calculated for the difference between the study sample and the excluded sample, using t-test for continuous variables and chi-square test of expected probabilities for categorical.

	Full Sample (*n* = 175)	Study Sample (*n* = 130)	Excluded (*n* = 45)	P
**Mean DNAm (%, sd)**				
All	50.81 (0.90)	50.80 (0.94)	50.85 (0.81)	0.73
Open Sea	73.63 (1.26)	73.61 (1.30)	73.69 (1.16)	0.71
Shelf	78.08 (1.37)	78.06 (1.40)	78.13 (1.30)	0.76
Shore	46.76 (0.85)	46.76 (0.87)	46.77 (0.81)	0.93
Island	19.41 (0.56)	19.40 (0.58)	19.44 (0.51)	0.65
Maternal Age (years)	33.30 (4.60)	33.62 (4.63)	32.36 (4.45)	0.11
**Sex**				
Female	85 (48.6%)	64 (49.2%)	21 (46.7%)	0.73
Male	90 (51.4%)	66 (50.8%)	24 (53.3%)	
**Maternal Education**				
High school or less	33 (18.9%)	26 (20.0%)	7 (15.6%)	
Less than 4 Yrs College	23 (13.1%)	18 (13.8%)	5 (11.1%)	
4 Yrs of College	77 (44.0%)	57 (43.8%)	20 (44.4%)	0.69
Post Graduate	35 (20.0%)	29 (22.3%)	6 (13.3%)	
Missing	7 (4.0%)	-	7 (16.6%)	
**Race**				
Non-Hispanic White	85 (48.6%)	66 (50.8%)	19 (42.2%)	
Non-Hispanic Black	15 (8.6%)	12 (9.2%)	3 (6.7%)	
Hispanic/Latino	23 (13.1%)	14 (10.8%)	9 (20.0%)	0.14
Other	48 (27.4%)	38 (29.2%)	10 (22.2%)	
Missing	4 (2.3%)	-	4 (8.9%)	
**Multi-vitamin Use**				
None Pre-Conception	102 (58.3%)	86 (66.2%)	16 (35.6%)	
Used Pre- Conception	51 (29.1%)	44 (33.8%)	7 (15.6%)	0.73
Missing	22 (12.6%)	-	22 (48.9%)	
**Prenatal Vitamin Use**				
None Pre- Conception	92 (52.6%)	79 (60.8%)	13 (28.9%)	
Used Pre- Conception	64 (36.6%)	51 (39.2%)	13 (28.9%)	0.26
Missing	19 (10.9%)	-	19 (42.2%)	
**Folic Acid Use**				
None Pre- Conception	131 (74.9%)	115 (88.5%)	16 (35.6%)	
Used Pre- Conception	18 (10.3%)	15 (11.5%)	3 (6.7%)	0.56
Missing	26 (14.9%)	-	26 (57.8%)	
**MTHFR C677T in child**				
Not Present	89 (50.9%)	63 (48.5%)	26 (57.8%)	
At least one allele	83 (47.4%)	67 (51.5%)	16 (35.6%)	0.08
Missing	3 (1.7%)	-	3 (6.7%)	
**MTHFR C677T in mom**				
Not Present	74 (42.3%)	55 (42.3%)	19 (42.2%)	
At least one allele	90 (51.4%)	75 (57.7%)	15 (33.3%)	0.11
Missing	11 (6.3%)	-	11 (24.4%)	
**Round**				0.004

1	115 (65.7%)	92 (70.8%)	23 (51.1%)	
2	60 (34.3%)	38 (29.2%)	22 (48.9%)	
**Cell Type (%, sd)**				
nRBC	10.1 (5.4)	9.8 (5.2)	11.0 (5.9)	0.23
Granulocytes	42.8 (12.5)	43.4 (12.3)	40.9 (12.9)	0.26

Supplementary Table S2. Folic acid use pre-conception by covariates. P-values for difference in variables by multivitamin intake is from t-test for continuous variables, and chi-square test for categorical variables.

	Folic Acid Pre-Conception		
	**No (*n* = 115)**	**Yes (*n* = 15)**	**P-value**
**Mean DNAm (%, sd)**			
All	50.8 (1.0)	51.0 (0.8)	0.31
Open Sea	73.6 (1.3)	73.9 (1.0)	0.21
Shelf	78.0 (1.4)	78.4 (1.1)	0.20
Shore	46.7 (0.9)	46.9 (0.8)	0.46
Island	19.4 (0.6)	19.5 (0.6)	0.58
Maternal Age (years)	33.6 (4.5)	33.9 (5.7)	0.82
**Sex:**			0.63
Female	58 (50.4%)	6 (40.0%)	
Male	57 (49.6%)	9 (60.0%)	
**Maternal Education**			0.71
High School or Less	23 (20.0%)	3 (20.0%)	
Less Than 4 Years College	15 (13.0%)	3 (20.0%)	
4 Years of College	52 (45.2%)	5 (33.3%)	
Post Graduate	25 (21.7%)	4 (26.7%)	
**Maternal Race**			0.70
Non-Hispanic White	58 (50.4%)	8 (53.3%)	
Non-Hispanic Black	12 (10.4%)	0 (0.00%)	
Hispanic/Latino	12 (10.4%)	2 (13.3%)	
Other	33 (28.7%)	5 (33.3%)	
**Multivitamins Pre-Conception**			0.81
No	77 (67.0%)	9 (60.0%)	
Yes	38 (33.0%)	6 (40.0%)	
**Prenatal Vitamins Pre-Conception**			0.36
No	72 (62.6%)	7 (46.7%)	
Yes	43 (37.4%)	8 (53.3%)	
**Child MTHFR C677T Genotype**			1.0
CC	56 (48.7%)	7 (46.7%)	
TT or TC	59 (51.3%)	8 (53.3%)	
**Maternal MTHFR C677T Genotype**			0.52
CC	47 (40.9%)	8 (53.3%)	
TT or TC	68 (59.1%)	7 (46.7%)	
**Round**			0.37
1	83 (72.2%)	9 (60.0%)	
2	32 (27.8%)	6 (40.0%)	
**Cell Type (%, sd)**			
nRBC	9.87 (5.35)	9.18 (3.80)	0.54
Granulocytes	43.3 (12.3)	44.5 (12.6)	0.72

Supplementary Table S3. Prenatal vitamin use pre-conception by covariates. P-values for difference in variables by multivitamin intake is from t-test for continuous variables, and chi-square test for categorical variables.

	Prenatal Vitamins Pre-Pregnancy		
	**No (n = 79)**	**Yes (n = 51)**	**P-value**
**Mean DNAm (%, sd)**			
All	50.8 (0.86)	50.7 (1.04)	0.50
Open Sea	73.7 (1.22)	73.5 (1.42)	0.31
Shelf	78.2 (1.30)	77.9 (1.55)	0.31
Shore	46.8 (0.81)	46.7 (0.95)	0.51
Island	19.4 (0.51)	19.4 (0.67)	0.55
Maternal Age (years)	33.4 (4.77)	34.0 (4.43)	0.50
**Sex:**			
Female	41 (51.9%)	23 (45.1%)	0.56
Male	38 (48.1%)	28 (54.9%)	
**Maternal Education**			
High School or Less	19 (24.1%)	7 (13.7%)	
Less Than 4 Years College	14 (17.7%)	4 (7.84%)	0.038
4 Years of College	27 (34.2%)	30 (58.8%)	
Post Graduate	19 (24.1%)	10 (19.6%)	
**Maternal Race**			
Non-Hispanic White	43 (54.4%)	23 (45.1%)	
Non-Hispanic Black	6 (7.59%)	6 (11.8%)	0.43
Hispanic/Latino	10 (12.7%)	4 (7.84%)	
Other	20 (25.3%)	18 (35.3%)	
**Multivitamins Pre-Conception**			
No	43 (54.4%)	43 (84.3%)	0.001
Yes	36 (45.6%)	8 (15.7%)	
**Prenatal Vitamins Pre-Conception**			
No	72 (91.1%)	43 (84.3%)	0.36
Yes	7 (8.86%)	8 (15.7%)	
**Child MTHFR C677T Genotype**			
CC	35 (44.3%)	28 (54.9%)	0.32
TT or TC	44 (55.7%)	23 (45.1%)	
**Maternal MTHFR C677T Genotype**			
CC	28 (35.4%)	27 (52.9%)	0.073
TT or TC	51 (64.6%)	24 (47.1%)	
**Round**			
1	57 (72.2%)	35 (68.6%)	0.82
2	22 (27.8%)	16 (31.4%)	
**Cell Type (%, sd)**			
nRBC	9.62 (5.09)	10.1 (5.36)	0.64
Granulocytes	44.0 (12.7)	42.5 (11.7)	0.49

Supplementary Table S4. Among DNA methylation sites nominally associated ($P<0.05$) with each exposure and genotype, number and proportion of sites that were hypermethylated and hypomethylated.

	DNA methylated sites hypomethylated		DNA methylated sites hypermethylated	
	N	**Percent**	***N***	**Percent**
**Multivitamin**				
Overall	3151	20.5	12191	79.5
Stratified by Maternal *MTHFR* C677T				
CC	1241	2.5	49076	97.5
TC/ TT	9772	75.7	3135	24.3
Stratified by Child *MTHFR* C677T				
CC	3215	9.8	29645	90.2
TC/TT	4932	57.6	3627	42.4
**Folic Acid**				
Overall	2467	14.3	14811	85.7
Stratified by Maternal *MTHFR* C677T				
CC	5605	7.7	66948	92.3
TC/TT	9425	63.6	5403	36.4
Stratified by Child *MTHFR* C677T				
CC	2985	18.9	12833	81.1
TC/TT	4299	39.2	6632	60.7
**Prenatal Vitamin**				
Overall	4199	39.2	6519	60.8
Stratified by Maternal *MTHFR* C677T				
CC	2515	23.9	8007	76.1
TC/TT	13265	74.9	4437	25.1
Stratified by Child *MTHFR* C677T				
CC	4683	50.2	4643	49.8
TC/TT	3820	34.8	7145	65.2
***MTHFR* C677T**				
TC/TT in Child	4913	5.6	83442	94.4
TC/TT in Mother	6299	7.3	80329	92.7

Supplementary Table S5. Top pathways for single site supplement models. Pathways with P<0.05 and at least 5 observed terms shown. For prenatal vitamins, pathways with at least 2 observed terms shown.

GO Term ID	GO Term Name	*N* Genes in Term	*N* Genes With CpG P<0.001	P	FDR
	**Multivitamins**				
GO:0007156	homophilic cell adhesion via plasma membrane adhesion molecules	151	14	1.90E-11	4.24E-07
GO:0022610	biological adhesion	1295	18	0.00077	1
GO:0007267	cell-cell signaling	1582	19	0.003	1
GO:0007399	nervous system development	2182	25	0.0031	1
GO:0007154	cell communication	6196	42	0.017	1
GO:0009416	response to light stimulus	288	5	0.018	1
GO:0023052	signaling	6172	41	0.029	1
	**Folic Acid**				
GO:0051056	regulation of small GTPase mediated signal transduction	312	10	0.00055	1
GO:1901700	response to oxygen-containing compound	1504	20	0.005	1
GO:0006955	immune response	1859	20	0.0052	1
GO:0001678	cellular glucose homeostasis	131	5	0.0057	1
GO:0050896	response to stimulus	8330	69	0.0075	1
GO:0043087	regulation of GTPase activity	445	9	0.011	1
GO:0006810	transport	4688	43	0.017	1
GO:0098657	import into cell	721	11	0.019	1
GO:0006897	endocytosis	646	10	0.02	1
GO:0007186	G-protein coupled receptor signaling pathway	1220	11	0.033	1
GO:0051707	response to other organism	794	9	0.033	1
GO:0009605	response to external stimulus	2082	21	0.041	1
GO:0009056	catabolic process	2397	23	0.046	1
GO:0009607	response to biotic stimulus	836	9	0.046	1
	**Prenatal Vitamins**				
GO:0016331	morphogenesis of embryonic epithelium	145	4	0.003	1
GO:0048486	parasympathetic nervous system development	17	2	0.0031	1
GO:0001504	neurotransmitter uptake	31	2	0.006	1
GO:0030224	monocyte differentiation	32	2	0.0061	1
GO:0031670	cellular response to nutrient	57	2	0.016	1
GO:0002181	cytoplasmic translation	78	2	0.024	1
GO:0050807	regulation of synapse organization	152	3	0.025	1
GO:0070509	calcium ion import	67	2	0.027	1
GO:0050808	synapse organization	282	4	0.028	1
GO:0050803	regulation of synapse structure or activity	163	3	0.029	1
GO:0043523	regulation of neuron apoptotic process	186	3	0.034	1

Supplementary Table S6. Top pathways for single site *MTHFR* C677T models. Pathways with p-value<0.001 and at least 5 terms shown.

GO Term ID	GO Term Name	*N* Genes in Term	*N* Genes With CpG P<0.001	P	FDR
	**Child *MTHFR***				
GO:0010811	positive regulation of cell-substrate adhesion	116	33	8.82E-07	0.01
GO:0031589	cell-substrate adhesion	324	60	0.00057	1
GO:0042310	vasoconstriction	70	17	0.00064	1
GO:0022610	biological adhesion	1336	192	0.00093	1
	**Maternal *MTHFR***				
GO:1901890	positive regulation of cell junction assembly	31	14	4.57E-05	0.23
GO:0051056	regulation of small GTPase mediated signal transduction	294	69	6.15E-05	0.23
GO:0010811	positive regulation of cell-substrate adhesion	116	31	0.000231	0.58
GO:0070647	protein modification by small protein conjugation or removal	970	153	0.000361	0.64
GO:0051704	multi-organism process	2229	307	0.000448	0.64
GO:0048012	hepatocyte growth factor receptor signaling pathway	16	8	0.000469	0.64
GO:0043412	macromolecule modification	3992	553	0.000556	0.64
GO:0051179	localization	6091	823	0.000577	0.64
GO:0051336	regulation of hydrolase activity	1208	190	0.000616	0.64
GO:0006464	cellular protein modification process	3801	531	0.000627	0.64
GO:0006810	transport	4689	635	0.000749	0.66
GO:0010923	negative regulation of phosphatase activity	95	24	0.000814	0.66
GO:0031589	cell-substrate adhesion	324	68	0.000855	0.66
GO:0007264	small GTPase mediated signal transduction	504	96	0.00093	0.66

Supplementary Table S7. GAMP test P-values for the effect of pre-conception supplement intake on cumulative density distribution of cord blood DNA methylation. Adjusted for sex, maternal age, maternal education, ancestry, batch, and cell type proportions for nucleated red blood cells and granulocytes.

	Overall	Open Sea	Shelf	Shore	Island
**Folic Acid**					
Unstratified	0.038	0.042	0.041	0.028	0.597
Stratified by Maternal *MTHFR*					
CC Genotype	0.095	0.087	0.097	0.089	0.686
CT or TT Genotype	0.131	0.139	0.155	0.127	0.315
Stratified by Child *MTHFR*					
CC Genotype	0.08	0.085	0.079	0.066	0.648
CT or TT Genotype	0.176	0.158	0.17	0.162	0.486
**Multivitamins**					
Unstratified	0.11	0.091	0.091	0.112	0.619
Stratified by Maternal *MTHFR*					
CC Genotype	0.029	0.039	0.042	0.016	0.056
CT or TT Genotype	0.369	0.337	0.351	0.34	0.432
Stratified by Child *MTHFR*					
CC Genotype	0.053	0.046	0.047	0.058	0.512
CT or TT Genotype	0.871	0.715	0.697	0.761	1.0
**Prenatal Vitamins**					
Unstratified	0.445	0.842	0.854	0.348	0.078
Stratified by Maternal *MTHFR*					
CC Genotype	0.358	0.388	0.356	0.297	0.436
CT or TT Genotype	0.581	1.0	0.831	0.729	0.192
Stratified by Child *MTHFR*					
CC Genotype	0.541	0.523	0.577	0.638	0.223
CT or TT Genotype	0.585	0.531	0.593	0.565	0.513

Supplementary Table S8. Global methylation differences by multivitamin supplementation prior to pregnancy, stratified by *MTHFR* genotype, in maternal blood and placenta.

	**Global Methylation Difference**	
**Tissue Type**	**CC Genotype**	**CT/TT Genotype**
Maternal Blood (Early Pregnancy)	-0.10 (-0.47, 0.26)	-0.08 (-0.40,0.23)
Maternal Blood (Late Pregnancy)	-0.28 (-1.03, 0.07)	-0.70 (-1.40, -0.01)
Placenta (Maternal Side)	0.57 (-0.30, 1.47)	0.35 (-1.21, 0.52)
Placenta (Fetal Side)	0.26 (-1.14, 1.66)	-0.17 (-0.61, 0.94)

Supplementary Figure S1. QQ-plots of single site models. A. Multivitamin model. B. Folic acid model. C. Prenatal vitamin model. D. Child *MTHFR* C677T model. E. Maternal *MTHFR* C677T model.

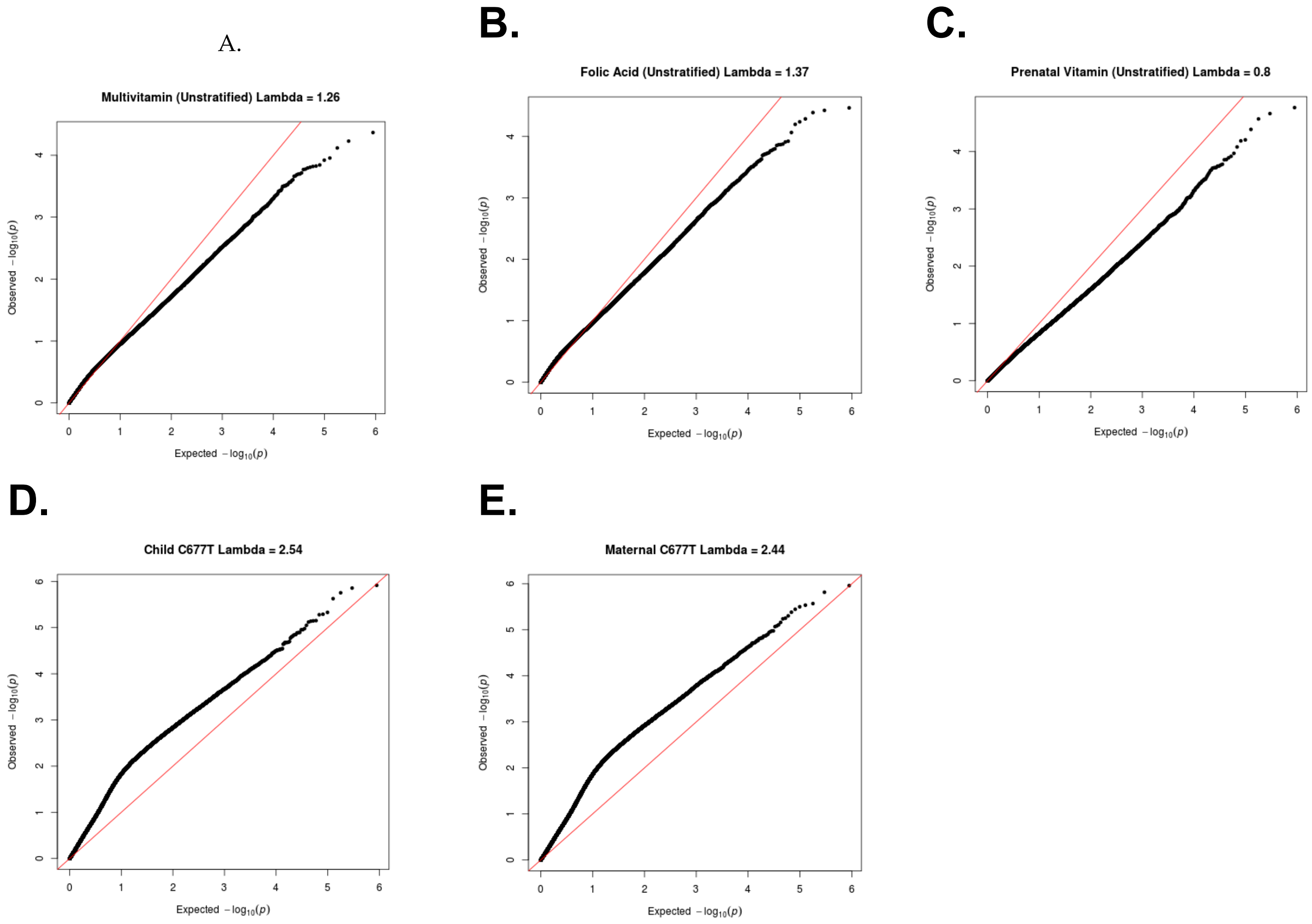

Supplementary Figure S2. Unadjusted supplement and global DNA methylation by genomic regions.

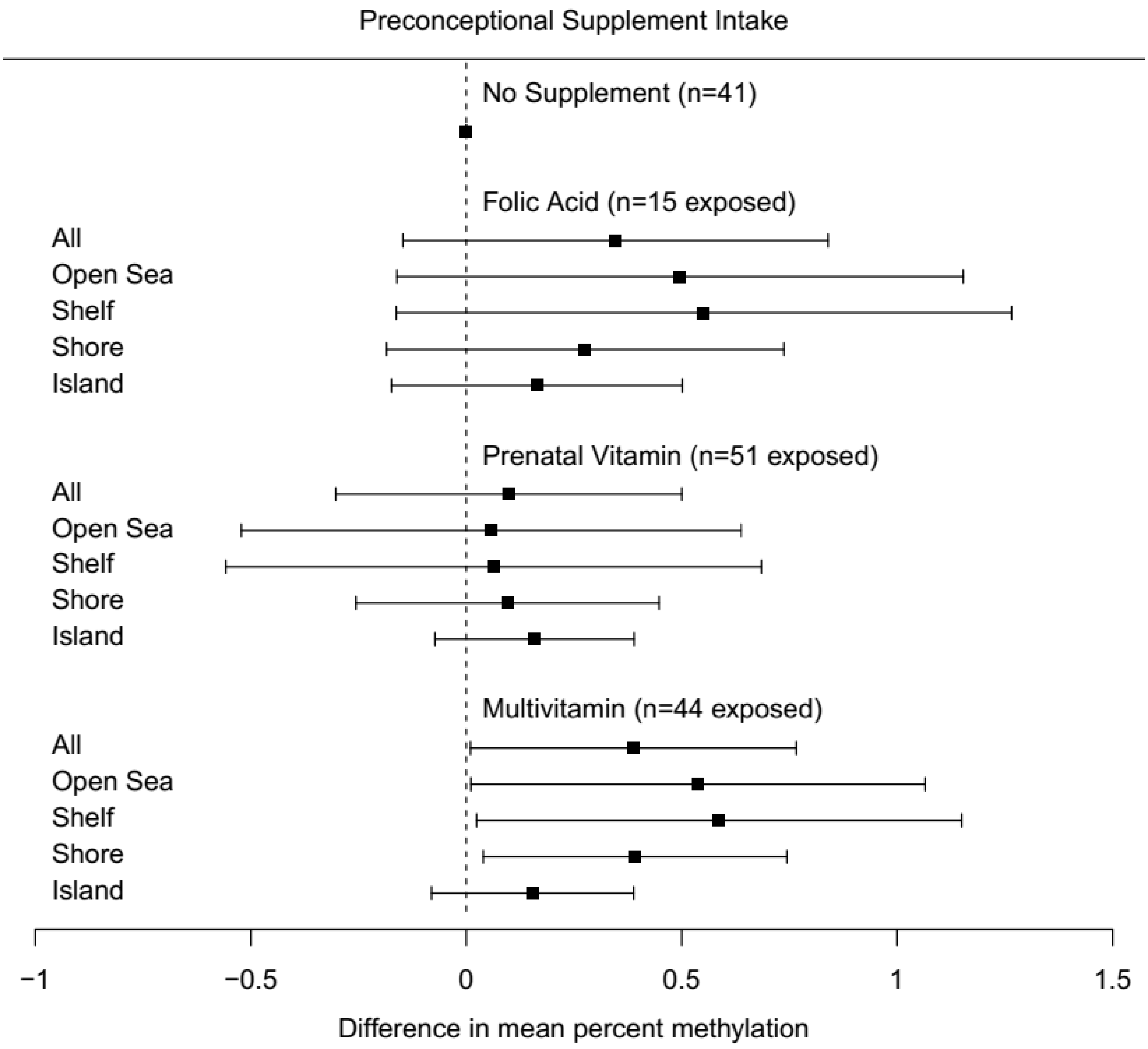

Supplementary Figure 3. Estimated model coefficients for effect of pre-conception folic acid use. Models were adjusted for sex, maternal age, maternal education, ancestry, batch, and cell type proportions for nucleated red blood cells and granulocytes. .

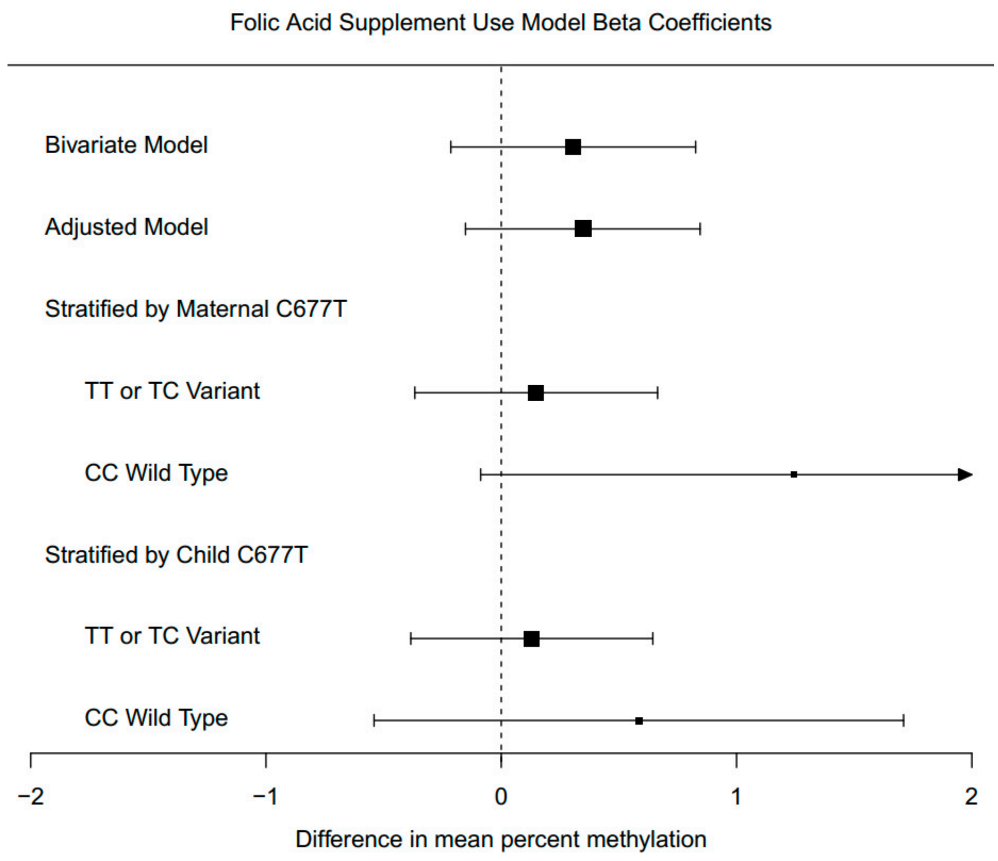

Supplementary Figure S4. Estimated model coefficients for effect of pre-conception prenatal vitamin use. Models were adjusted for sex, maternal age, maternal education, ancestry, batch, and cell type proportions for nucleated red blood cells and granulocytes. .

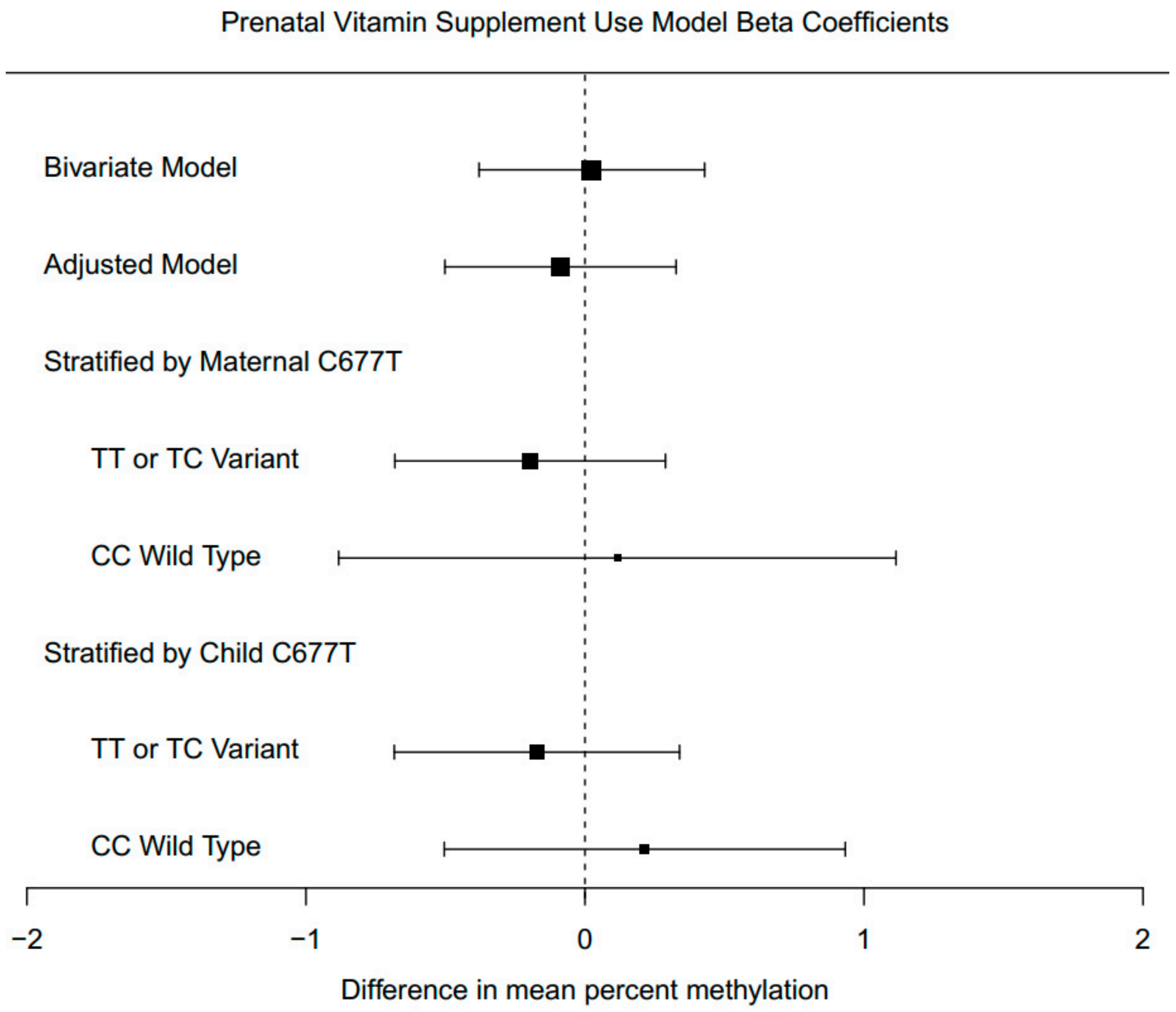

Supplement: Supplementary file 1 [file ijerph-17-09190-s001.pdf]
